# Supplementary figures and images for: Identification of primary genes in glomeruli compartment of immunoglobulin A nephropathy by bioinformatic analysis
Source: PeerJ. 2019 Jul 19;7:e7067. doi: 10.7717/peerj.7067 (PMC6645034; doi:10.7717/peerj.7067)

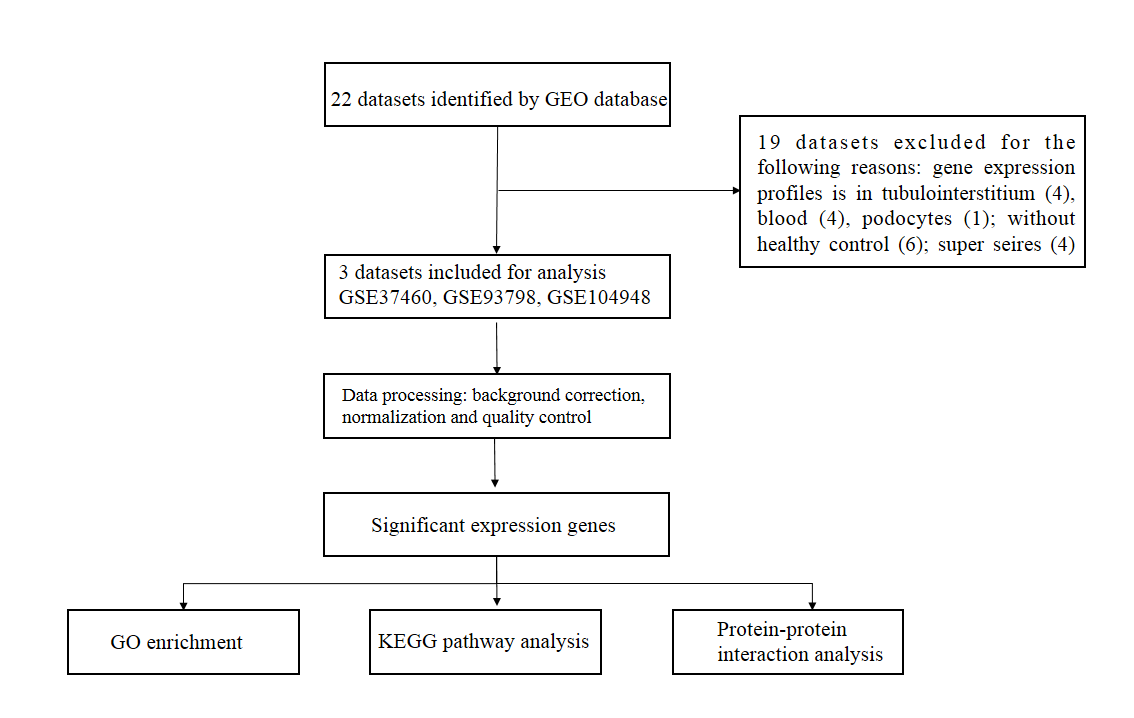

Supplement: Figure S1 [file peerj-07-7067-s001.png]
