# Supplementary material for: Identification of primary genes in glomeruli compartment of immunoglobulin A nephropathy by bioinformatic analysis
Source: PeerJ. 2019 Jul 19;7:e7067. doi: 10.7717/peerj.7067 (PMC6645034; doi:10.7717/peerj.7067)
Supplement: Table S1 [file peerj-07-7067-s002.docx]

|  | IQC | EQC | CQCg | CQCp | AQCg | AQCp | SMR |
| --- | --- | --- | --- | --- | --- | --- | --- |
| GSE37460 | 20 | 2 | 14.85 | 1.27 | 6.99 | 0.29 | 1.17 |
| GSE93798 | 4 | 2 | 6.74 | 0.91 | 3.69 | 0.1 | 2.17 |
| GSE104948 | 2.67 | 2 | 8.15 | 0.76 | 1.91 | 0.03 | 2.67 |

IQC, internal quality control; EQC, external quality control; CQCg, consistency quality control for genes; CQCp, consistency quality control for pathways; AQCg, accuracy quality control for genes; AQCp, accuracy quality control for pathways; SMR, standardized mean rank.
